# Calvarial venous malformation highlighted by the flow–reflux phenomenon on ultrasound

**DOI:** 10.5334/jbsr.4220

**Published:** 2026-03-06

**Authors:** Anas Messaoudi, Lokmane Taihi, Jacques Malghem

**Affiliations:** 1 Department of radiology, Cliniques Universitaires Saint‑Luc, Brussels, Belgium

**Keywords:** calvarium, intraosseous hemangioma, venous malformation, ultrasound, Doppler, flow–reflux phenomenon, low‑flow vascular malformation

## Abstract

A case is reported of a woman presenting with a firm, tender right frontal lump. Ultrasound showed a small, irregular subgaleal mass with tiny bone surface perforations. Color Doppler ultrasound demonstrated compression–decompression induced alternating flow signals, indicating flow and reflux through the bone lesion. CT revealed an expansile osteolytic frontal lesion with a honeycomb pattern, consistent with an intraosseous venous malformation, which was confirmed histologically. The added value of compression–decompression color Doppler ultrasound in low‑flow calvarial vascular malformations is illustrated.

*Teaching point:* Compression–decompression color Doppler ultrasound can reveal flow–reflux in low‑flow intraosseous venous malformations, contributing to the diagnosis.

## Case Presentation

A 48‑year‑old woman presented with a firm swelling in the right frontal region, present for two years. The lesion was slightly tender on palpation and the overlying skin appeared normal. There was no history of trauma or prior surgery.

**Figure 1:**
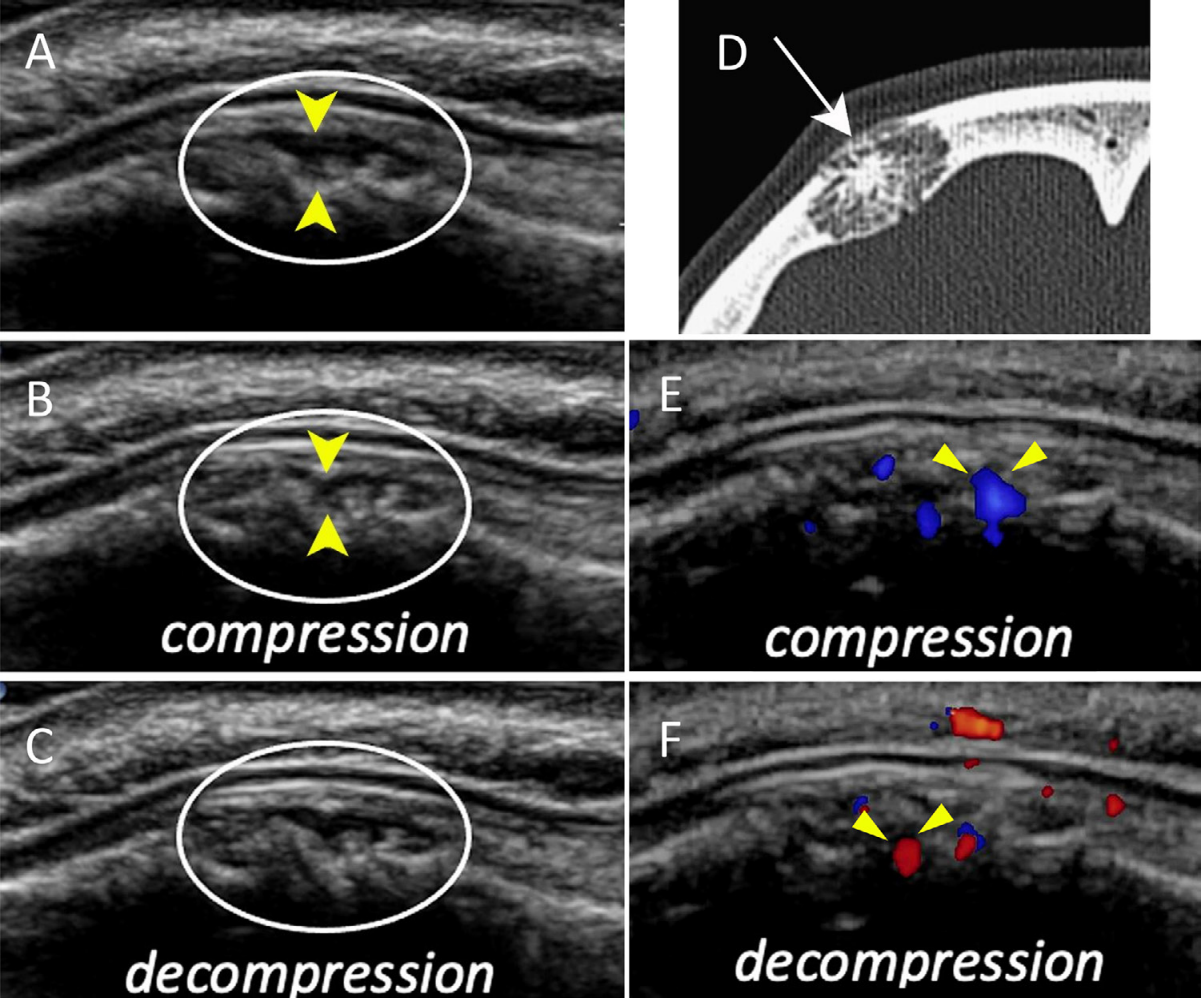
Calvarial venous malformation of the right frontal bone. **A:** Ultrasound image showing hypoechoic thickening of the right frontal calvarium with irregular cortical surface (yellow arrowheads). **B, C:** Ultrasound during compression **(B)** and decompression **(C)** demonstrating compressible cavities (yellow arrowheads). **D:** CT image showing an expansile osteolytic lesion of the right frontal bone with radiating trabeculae, creating a sunburst/honeycomb appearance. **E, F:** Color Doppler ultrasound during compression **(E)** and decompression **(F)** demonstrating a flow–reflux phenomenon with inversion of flow color during ther maneuver (arrowheads).

Ultrasound revealed a small, heterogeneous, hypoechoic nodular mass beneath the galea overlying a focal irregular surface of the underlying frontal bone (Figure 1A). The mass was slightly compressible (Figures 1B and 1D). No vascular signal was observed on color Doppler imaging. However, dynamic assessment with gentle compression with the transducer induced forward flow within the cavities, and vascular reflux during decompression (Figures 1E and 1F). This flow–reflux phenomenon was interpreted as suggestive of a low‑flow vascular malformation.

Computed tomography (CT) confirmed an expansile osteolytic lesion of the right frontal bone, composed of thin radiating trabeculae creating a characteristic sunburst appearance (Figure 1D). These imaging findings were consistent with an intraosseous vascular malformation, which was confirmed histologically after resection.

## Discussion

Vascular bone lesions can be present in adults, but the terminology is often misleading. In particular, the term hemangioma is often used inappropriately, and venous malformations are frequently referred to as hemangiomas or cavernous hemangiomas [1, 2]. A similar misclassification occurs in the calvarium, where the term intraosseous hemangioma is often applied, although the term low‑flow vascular malformation or venous malformation would be preferable. CT is considered the imaging modality of choice, as it typically demonstrates characteristic trabecular patterns, such as a honeycomb or sunburst appearances [3].

Although ultrasound is not routinely used for the evaluation of bone lesions, it can provide valuable information when an extraosseous component is present and accessible. In such cases, ultrasound allows real‑time assessment of lesion compressibility and vascular behavior [4]. The demonstration of compressible vascular spaces and, most convincingly, the dynamic flow–reflux phenomenon on Doppler imaging is highly suggestive of low‑flow vascular malformations. This dynamic sign reflects slow venous drainage and low intralesional pressure, with hemodynamics resembling spongy vascular tissue rather than a solid tumor.

Dynamic color Doppler analysis may suggest the diagnosis of intraosseous vascular malformation by demonstrating the flow–reflux phenomenon. When combined with CT confirmation of characteristic trabecular bone changes, this multimodal approach enables a confident, non‑invasive diagnosis and contributes to selecting the appropriate therapeutic strategy.

## References

[r1] Marcelin C, Dubois J, Kokta V, et al. Soft tissue vascular tumor‑like lesions in adults: Imaging and pathological analysis pitfalls per ISSVA classification. Insights Imaging. 2024;15(1):135. 10.1186/s13244-024-01712-w. PMID: ; PMCID: . 38853199 PMC11162993

[r2] Wassef M, Vanwijck R, Clapuyt P, Boon L, Magalon G. Tumeurs et malformations vasculaires, classification anatomopathologique et imagerie [Vascular tumours and malformations, classification, pathology and imaging]. Ann Chir Plast Esthet. 2006;51(4–5):263–281. 10.1016/j.anplas.2006.07.017. Epub 2006 Sep 26. PMID: . 17005309

[r3] Garfinkle J, Melançon D, Cortes M, Tampieri D. Imaging pattern of calvarial lesions in adults. Skeletal Radiol. 2011;40(10):1261–1273. 10.1007/s00256-010-0971-8. Epub 2010 Jun 6. PMID: . 20526773

[r4] Malghem J, Taihi L, Kirchgesner T, et al. Le flux et le reflux en Doppler couleur dans les malformations veineuses. In: Campagna R, Faruch‑Bilfeld M, Jacob D, Meyer P, Ponsot A, eds. Actualités en échographie de l’appareil locomoteur. Sauramps Médical; 2024: 155–164.

